# Chronic multimorbidity impairs role functioning in middle-aged and older individuals mostly when non-partnered or living alone

**DOI:** 10.1371/journal.pone.0170525

**Published:** 2017-02-02

**Authors:** Fabiola Müller, Mariët Hagedoorn, Marrit A. Tuinman

**Affiliations:** Department of Health Psychology, University of Groningen, University Medical Center Groningen, Antonius Deusinglaan 1, Groningen, The Netherlands; Texas Technical University Health Sciences Center, UNITED STATES

## Abstract

**Background:**

Due to the aging of the population, society includes a growing proportion of older individuals prone to chronic morbidity. This study aimed to investigate the adverse effects of single and multiple chronic morbidity on psychosocial health and whether these effects are more pronounced in individuals who are non-partnered or living alone.

**Materials and methods:**

Baseline data from the ‘Lifelines Cohort Study’ collected between 2006 and 2013 in the Netherlands were used. Individuals aged 50+ (n = 25,214) were categorized according to their health status (healthy, single chronic morbidity, multiple chronic morbidity), relationship status (partnered, non-partnered), and living arrangement (living with someone, living alone). Analyses of covariance (ANCOVA) were performed to study the main- and the interaction-effects on mental health and role functioning as assessed with the RAND-36.

**Results:**

Irrespective of having chronic morbidity, having a partner was associated with better mental health when partners shared a home. Individuals with single and especially multiple chronic morbidity had impaired role functioning. Having a partner mitigated the adverse effects of multimorbidity on role functioning, but only in individuals who shared a home with their partner. Non-partnered individuals with multimorbidity and those not sharing a home with their partner demonstrated impaired role functioning.

**Conclusions:**

The results demonstrate that multimorbidity negatively affects role functioning, but not the mental health, of middle-aged and older individuals. Sharing a home with a partner can mitigate these adverse effects, while other combinations of relationship status and living arrangement do not. Offering intervention to those individuals most vulnerable to impaired functioning may relieve some of the increasing pressure on the health care system. An individual’s relationship status along with one’s living arrangement could foster the identification of a target group for such interventions attempting to sustain physical functioning or to adapt daily goals.

## Introduction

Due to the aging of the population, society is confronted with a growing proportion of individuals suffering from single and multiple chronic morbidity. At the same time, a substantial number of older individuals do not have a partner or live on their own. As is the case with having a chronic disease, being non-partnered and living alone have been associated with poorer psychosocial health outcomes. This study aimed to investigate whether individuals with single and multiple chronic morbidity are especially vulnerable to impaired mental health and role functioning when they are non-partnered or live alone. By identifying and targeting those individuals who have the greatest need for additional care, the increasing pressure on the health care system could be reduced.

Chronic morbidities are common in middle-aged and older individuals and may have serious adverse effects on psychosocial health. Approximately half of individuals aged 50 years and older, and up to 70% of those aged 65–74 years, suffer from at least one chronic condition [1,2]. Multimorbidity, which is the accumulation of multiple (chronic) diseases within one individual [3], has been estimated to occur in 55% and up to 98% of older individuals [4]. Several studies suggest that chronic diseases are related to reduced psychosocial health. For example, individuals with chronic diseases such as type 2 diabetes [5], rheumatoid arthritis [6], and hypertension [7] experienced impaired mental health and physical functioning compared to the general population and normotensive individuals, respectively. These psychosocial impairments appear even more severe in individuals with multiple chronic morbidity. An increasing number of chronic diseases within one individual is associated with declining quality of life, decreased physical functioning and an increasing prevalence of mental health problems (i.e., depression, anxiety) [2,4,8–10].

These adverse effects of chronic morbidity on psychosocial health might be even more severe for individuals who do not have an intimate partner. Previous research has demonstrated that, in general, individuals who are non-partnered have poorer mental health than their partnered peers [11–13], likely because they lack support [14,15]. While this evidence mainly comes from studies on populations without a specific medical diagnosis, non-partnered, chronically ill individuals may be especially vulnerable to psychosocial health problems because they have to face ongoing challenges related to their disease (e.g., distressing symptoms, daily disease management tasks) without a partner. Research on the associations between an individual’s relationship status and psychosocial health among (chronically) ill individuals is scarce, but indeed indicates that non-partnered patients experience poorer psychosocial health compared to partnered patients [16,17]. Because suffering from multiple chronic diseases can be even more burdensome, non-partnered individuals with multimorbidity might be even more vulnerable to poor psychosocial health.

In addition to having a partner, whether one lives alone or shares a home with someone, may also determine a person’s vulnerability to poor psychosocial health when chronically ill. In middle-aged and older individuals without a specific medical diagnosis, those who live alone or live with someone other than a partner have been found to report more depressive symptoms, impaired physical functioning, and more distress than individuals living with a partner [18–20]. Research on older individuals in a LAT [living apart together]-relationship, which is having a partner but not sharing a home, is still scarce, but indicates that these individuals are less happy and receive less support compared to partnered individuals sharing a home [21,22]. Additionally, research is scarce on the psychosocial health effects of an individual’s relationship status and living arrangement in the vulnerable group of older individuals with chronic morbidity. There is some evidence that cancer patients in a LAT-relationship are as highly distressed as non-partnered patients [16] and that patients with various chronic diseases have poorer mental health, but not physical functioning, when living in an arrangement other than with a partner [23]. These studies indicate that the benefit of having a partner depends on sharing a home and that being partnered and sharing a home is the most beneficial combination of relationship status and living arrangement. However, the interaction-effects of relationship status and living arrangement have not been investigated systematically and have rarely been considered for the vulnerable group of individuals with either single or multiple chronic morbidity. Therefore, the present study aims to investigate whether middle-aged and older individuals with single and multiple chronic morbidity are especially susceptible to impaired mental health and role functioning when they are non-partnered or living alone. The following three hypotheses were investigated:

**Hypothesis 1:** Middle-aged and older individuals with single and especially multiple chronic morbidity experience impaired mental health and role functioning compared to healthy individuals.

**Hypothesis 2:** An individual’s relationship status moderates the effect of chronic morbidity on mental health and role functioning: non-partnered individuals experience worse mental health and role functioning than partnered individuals. The interaction-effect is expected to be most pronounced in individuals with multiple chronic morbidity.

**Hypothesis 3:** An individual’s living arrangement moderates the effect of an individual’s relationship status on mental health and role functioning: Partnered individuals are expected to benefit in terms of mental health and role functioning only when sharing a home with their partners. Non-partnered individuals are not expected to benefit in terms of mental health and role functioning when sharing their home with someone. The interaction-effects are expected to be most pronounced in individuals with multiple chronic morbidity.

## Materials and methods

### Procedure

The current research draws on the baseline data from the ‘Lifelines Cohort Study’ collected between November 2006 and December 2013. Lifelines is a multi-disciplinary, prospective, population-based cohort study examining in a unique three-generation design the health and health-related behaviors of 167,729 persons living in the northeast region of the Netherlands. It employs a broad range of investigative procedures in assessing the biomedical, socio-demographic, behavioral, physical, and psychological factors that contribute to the health and disease of the general population, with a special focus on multimorbidity and complex genetics. Lifelines is a facility that is open for all researchers. Information on application and data access procedure is summarized on www.lifelines.net.

A random sample of individuals between 25 and 50 years old was recruited with a letter from their General Practitioner [GP]. Individuals were not invited if the GP considered the person ineligible based on three exclusion criteria: (1) presence of a severe psychiatric or physical illness, (2) limited life expectancy (< 5 years), and (3) insufficient knowledge of the Dutch language. Additionally, family members (including the partner) of enrolled participants were recruited after permission was provided by the participant to invite his or her relatives. Finally, interested individuals who were not approached by their GP or via family members could enroll themselves on the Lifelines website. Individuals interested in participating received information on the Lifelines study and the informed consent form. After the informed consent form was signed and returned to the Lifelines Research Site, participants received the first part of the baseline-questionnaire by mail. During the first visit to the Lifelines Research Site, the first part of the questionnaire was checked for completeness and the second part of the questionnaire was provided and was later checked during the second visit to the Lifelines Research Site. The goal was to plan the two visits in short succession within several weeks. The Lifelines study was approved by the medical ethical committee of the University Medical Center Groningen [UMCG] in the Netherlands and was conducted in accordance with the Declaration of Helsinki and the research code UMCG. More detailed information about the recruitment and study design of Lifelines can be found elsewhere [24].

### Participants

Baseline data were available from 48,263 participants aged 50 years or older (total adult sample n = 152,180). First, one partner from a participating couple was randomly excluded to avoid dependency in the data (n = 11,752, 24.3%). Furthermore, we excluded participants with severe cognitive/psychiatric conditions (e.g., Parkinson’s or Alzheimer’s disease; n = 697, 1.4%), participants who could not fill in the questionnaire themselves (proxy-questionnaire), or those who reported living in a nursing home or residential care home (n = 2,012, 4.2%). Participants who had a long time gap between filling in the two parts of the baseline-questionnaire were also excluded (> 6 months, n = 123, 0.3%). Participants whose health status, relationship status, or living arrangement could not clearly be identified due to inconsistent or missing answers and participants who were partnered and living with someone other than that partner were excluded as well (n = 515, 1.1%). Finally, we excluded participants with conditions that may have a wide range of severity or conditions in which case severity and treatment were unknown in our sample (e.g., asthmatic disease, migraine; n = 7,950, 16.5%).

### Measures

#### Sociodemographics and independent measures

Sociodemographic variables included age, gender, level of education, and occupational status.

The participant’s health status was assessed by means of self-report (e.g., ‘Do you have heart failure (decreased pumping of the heart)?’) with answer categories including ‘yes’, ‘no’, and ‘I don’t know’. For several conditions, the type of treatment (e.g., ‘If you have diabetes, how are you being treated?’), whether the condition was ever officially diagnosed (e.g., ‘Was the arrhythmia ever diagnosed by a doctor and/or in the hospital?’), the time of onset (e.g., ‘Since which year do you have cancer?’), and severity (e.g., ‘If you have ever been diagnosed with disturbed kidney function, do you know the severity of the disorder?’) were surveyed. Other conditions were presented as a checklist (e.g., Multiple Sclerosis, Fibromyalgia, Crohn’s disease). The following three health status groups were identified: (a) healthy, (b) single chronic morbidity (single CM), and (c) multiple chronic morbidity (multiple CM). To define the health status groups ‘single CM’ and ‘multiple CM’, we aimed to include chronic conditions that have an impact on daily life by requiring daily disease management (e.g., exercise, (self-)medication, diet) or through interfering symptoms (e.g., pain, stiffness). Table 1 provides an overview of the chronic conditions included, their operationalization, and their frequency in the sample. Participants suffering from conditions with unknown or varying severity, or with unknown or varying need for treatment (e.g., asthmatic diseases, migraine, hepatitis, thrombose) were not categorized as ‘single CM’ or ‘multiple CM’ unless they also suffered from one or more of the target chronic conditions as presented in Table 1. Participants were categorized as ‘healthy’ if they were neither suffering from any of the target chronic conditions nor from a condition with unknown severity or need for treatment. Because the sample consists of middle-aged and older individuals, participants with conditions that were not assumed to have a profound impact on daily life (e.g., allergies, cataract, gallstones, skin conditions such as eczema) were permitted in the ‘healthy’ group to foster generalizability. Participants could also provide a free description of any other condition from which they suffered that was not included in the questionnaire. For those participants who were categorized as being ‘healthy’, we searched those descriptions for relevant conditions by key terms and re-categorized participants if necessary.

**Table 1:** Selection and operationalization of chronic morbidity and its distribution across the sample.

Chronic Morbidity	Operationalization	Total Sample		Single CM		Multiple CM	
		n = 25,214		n = 9,686		n = 7,171	
		n^c^	%^c^	n	%	n^c^	%^c^
**Cancer**	Yes, recent (< = 2 years), serious type (excl. Basal Cell	114	0.5%	51	0.5%	63	0.9%
	Carcinoma), not cured (yet)						
**Cardiovascular Disease**							
Atherosclerosis	Checklist item	383	1.5%	33	0.3%	350	4.9%
Cardiac Arrhythmia	Yes, official diagnosed by physician and/or hospital,	1,730	6.9%	332	3.4%	1,398	19.5%
	in treatment^a^ (drugs, pacemaker)						
Heart Failure	Yes, in treatment^a^ (diet, drugs, pacemaker, transplant)	379	1.5%	25	0.3%	354	4.9%
High Cholesterol	Yes, in treatment^a^ (diet, drugs)	4,919	19.5%	1,233	12.7%	3,686	51.4%
Hypertension	Yes, in treatment^a^ (diet, drugs)	7,432	29.5%	2,779	28.7%	4,653	64.9%
Stroke	Yes	421	1.7%	61	0.6%	360	5.0%
**Multiple Sclerosis**	Checklist item	75	0.3%	44	0.5%	31	0.4%
**Diabetes Mellitus**	Yes, Diabetes type 1 or 2, in treatment^a^ (diet, drugs,	1,392	5.5%	199	2.1%	1,193	16.6%
	insulin)						
**Gastro Intestinal Disease**							
Coelic Disease	Checklist item	100	0.4%	51	0.5%	49	0.7%
Crohn’s Disease	Checklist item	109	0.4%	40	0.4%	69	1.0%
Liver Cirrhosis	Checklist item	45	0.2%	16	0.2%	29	0.4%
Ulcerative Colitis	Checklist item	225	0.9%	102	1.1%	123	1.7%
**Musculoskeletal Disease**							
Arthritic Disease	Checklist: Osteoarthritis, Rheumatoid Arthritis	6,680	26.5%	3,267	33.7%	3,413	47.6%
	Free description: Bechterew's Disease, Gout						
Fibromyalgia	Checklist item	1,571	6.2%	465	4.8%	1,106	15.4%
**Pulmonary Disease**							
COPD, Chronic Bronchitis,	Yes	2,409	9.6%	977	10.1%	1,432	20.0%
Emphysema							
**Renal Disease**^**b**^		82	0.3%	11	0.1%	71	1.0%
Disturbed Kidney Function	Yes, severely disturbed functioning, still present						
Kidney Disease	Yes, in treatment^a^ (drugs, diet, dialysis, transplant)						
Kidney Transplant	Yes						

^a^ Participants reported receiving at least one of the listed treatments.

^b^ The three renal diseases are combined into one illness group (i.e., an individual reporting to have a kidney transplant and a disturbed kidney function would not be considered as suffering from multiple chronic morbidity).

^c^ Due to multimorbidity in our sample, the number and percentage of participants per disease category do not add up to the total group number and 100%, respectively.

Relationship status was assessed by two different items due to a change of questionnaire-items during data-collection (‘What is your marital status?’ and ‘Do you currently have a partner?’). If the provided answer categories were not sufficient, participants could fill in a free description of their relationship status. We dichotomized the answers into (a) partnered (married, cohabiting, LAT-relationship), or (b) non-partnered (single, divorced, widowed).

Living arrangement was assessed with several items. First, participants could indicate whether they share a home (‘Please indicate whether the following people live with you in your home (more than half of the time), or not, or that you do not have this person (anymore).’). Eight options were provided e.g., ‘my partner’, ‘my father’, ‘child(ren)’. Responses included ‘yes’, ‘no’, and ‘I don’t have this person (anymore)’. Finally, participants could indicate if they live alone (‘I live alone’, ‘yes’, ‘no’). To validate these items, we used the item assessing the number of individuals living in the participant’s household (‘How many people live in your home, including yourself?’). With these items, we categorized participants as either (a) living with someone or (b) living alone.

#### Dependent measures

The subscale ‘mental health’ of the RAND-36 assesses psychological distress and well-being in the four weeks preceding the questionnaire with five items (e.g., ‘Have you felt downhearted and blue?’, ‘Have you felt calm and peaceful?’). Answers are scored on a 6-point Likert scale ranging from ‘all of the time’ to ‘none of the time’ [25,26]. Negatively formulated items were recoded. The scale-score was transformed into a 100-point-scale with higher scores indicating better mental health. The Dutch translation of the RAND-36 shows good psychometric qualities [27]. Reliability in this study was good, Cronbach’s α = .83.

The subscale ‘role functioning physical’ of the RAND-36 was used to assess role functioning. Four items assess limitations experienced in daily life or work due to physical problems in the past four weeks (e.g., ‘Cut down the amount of time you spent on work or other activities’, ‘Accomplished less than you would like’). Participants could respond either ‘yes’ or ‘no’. The scale-score was transformed into a 100-point-scale with higher scores indicating better role functioning [27]. Reliability in this study was good, Cronbach’s α = .90.

### Statistical analyses

A Chi-square test and analyses of variance were used to preliminary investigate potential covariates. For the primary analysis, two analyses of covariance (ANCOVA) were performed to determine the main- and interaction-effects of health status (healthy, single CM, multiple CM), relationship status (partnered, non-partnered), and living arrangement (living with someone, living alone) on mental health and role functioning. To interpret significant interaction-effects, simple effects analyses were performed. Cohen’s d is used to report effect sizes.

## Results

### Sample description

Our working sample consisted of 25,214 participants. Thirty-three percent (n = 8,357) of the participants were categorized as ‘healthy’, 38.4% (n = 9,686) as ‘single CM’ and 28.4% (n = 7,171) as ‘multiple CM’. Of those participants suffering from a single chronic morbidity, most were diagnosed with arthritic disease (n = 3,267, 33.7%) or hypertension (n = 2,779, 28.7%). Of the participants with multiple chronic morbidity, 61% (n = 4,376) suffered from two conditions, 26.2% (n = 1,877) suffered from three conditions and 9.4% (n = 672) suffered from four conditions. The remaining individuals (3.4%, n = 246) suffered from up to nine chronic conditions. Most individuals were categorized as either partnered (n = 21,384, 84.8%) or living with someone (n = 21,365, 84.7%). Of the partnered individuals, the large majority reported living with their partners (n = 20,775, 97.2%). Of the non-partnered individuals, 15.4% (n = 590) shared their homes, mostly with their children (n = 496, 84.1%). Some were living with siblings (n = 35, 5.9%), other adults (e.g., uncle, aunt, grandparent, friend; n = 35, 5.9%) or their mother (n = 34, 5.8%). The sample characteristics are summarized in Table 2.

**Table 2:** Sample characteristics.

Characteristics	Total Sample	Health Status		
		Healthy	Single CM	Multiple CM
	n = 25,214	n = 8,357	n = 9,686	n = 7,171
**Gender, female**	14,981 (59.4%)	4,438 (53.1%)	6,071 (62.7%)	4,472 (62.4%)
**Age** in years (mean, SD)	59.5 (7.6)	56.9 (6.7)	59.5 (7.4)	62.5 (7.8)
Range	50–93	50–87	50–89	50–93
**Educational Level**				
Low	6,561 (26%)	1,829 (21.9%)	2,443 (25.2%)	2,289 (31.9%)
Medium	11,939 (47.7%)	3,971 (47.5%)	4,663 (48.1%)	3,305 (46.1%)
High	5,752 (22.8%)	2,311 (27.7%)	2,194 (22.7%)	1,247 (17.4%)
Other or unknown	962 (3.8%)	246 (3%)	386 (3.9%)	330 (4.6%)
**Occupational Status**				
Employed (full time or part time)	14,015 (55.6%)	5,952 (71.2%)	5,382 (55.6%)	2,681 (37.4%)
Unable to work	1,194 (4.7%)	127 (1.5%)	434 (4.5%)	633 (8.8%)
Retired	6,046 (24%)	1,283 (15.4%)	2,281 (23.5%)	2,482 (34.6%)
Unemployed	696 (2.8%)	203 (2.4%)	273 (2.8%)	220 (3.1%)
Other or unknown	3,263 (13%)	792 (9.5%)	1,316 (13.6%)	1,155 (16.1%)
**Relationship Status, partnered**	21,384 (84.8%)	7,315 (87.5%)	8,193 (84.6%)	5,876 (81.9%)
**Living Arrangement, living with someone**	21,365 (84.7%)	7,354 (88.0%)	8,182 (84.5%)	5,829 (81.3%)

Note. Unless otherwise indicated, n and % are given; CM = Chronic Morbidity. Of the total sample (n = 25,214), most participants (56.1%) were recruited through family members, 24.7% enrolled themselves for participation on the Lifelines website, and the remaining 19.2% were recruited by their GP.

### Preliminary results

Preliminary analyses revealed that the distribution across the independent factors (health status, relationship status, and living arrangement) differed as a function of gender, χ²(2, N = 25,214) = 206.6, p < .001; χ²(1, N = 25,214) = 540.1, p < .001; χ²(1, N = 25,214) = 368.2, p < .001, and age, F(2, 25,211) = 1,145.0, p < .001; F(1, 25,212) = 488.6, p < .001; F(1, 25,212) = 854.0, p < .001. Similarly, mean scores of mental health, F(1, 25,207) = 785.1, p < .001, and role functioning, F(1, 25,176) = 157.5, p < .001, differed for men and women, with men having better psychosocial health than women. Hence, we controlled for gender and age in our analyses. The tested models, controlling for gender and age, for mental health, F(13, 25,195) = 141.3, p < .001, and role functioning, F(13, 25,164) = 142.4, p < .001, were significant. As is common in large datasets, Levene’s test indicated that the assumption of homogeneity of variances had been violated. Therefore, data analyses (ANCOVAs) with bootstrapping (based on 1000 bootstrap samples) were performed and yielded the same results as those presented here. The corrected ANCOVA models explained 6.8% and 6.9% (partial eta squared) of the variance in mental health and role functioning, respectively. See Table 3 for descriptive statistics and Table 4 for an overview of the main- and interaction-effects.

**Table 3:** Descriptive statistics for the outcomes of mental health and role functioning.

Health Status	Relationship Status	Living Arrangement	Mental Health				Role Functioning			
			Mean (SD)	n^a^	CI Lower^b^	CI Upper^b^	Mean (SD)	n^a^	CI Lower^b^	CI Upper^b^
**Healthy**	**partnered**	**with someone**	83.9 (13.5)	7,114	83.6	84.2	94.3 (31.7)	7,110	93.6	95.0
		**alone**	80.9 (13.2)	200	79.1	82.7	94.2 (31.1)	199	89.9	98.6
	**non-partnered**	**with someone**	79.4 (13.3)	239	77.7	81.0	91.0 (31.2)	239	87.1	95.0
		**alone**	79.2 (13.2)	803	78.3	80.2	91.7 (31.1)	801	89.5	93.8
**Single CM**	**partnered**	**with someone**	81.6 (13.3)	7,955	81.3	81.9	84.7 (31.3)	7,946	84.0	85.4
		**alone**	79.2 (13.2)	236	77.5	80.9	84.6 (31.0)	236	80.6	88.6
	**non-partnered**	**with someone**	75.4 (13.3)	225	73.6	77.1	80.8 (31.1)	225	76.7	84.8
		**alone**	75.8 (13.4)	1,268	75.1	76.5	80.2 (31.6)	1,265	78.4	81.9
**Multiple CM**	**partnered**	**with someone**	79.6 (13.4)	5,703	79.3	80.0	74.5 (31.6)	5,690	73.7	75.3
		**alone**	75.3 (13.2)	173	73.4	77.3	66.5 (31.1)	173	61.9	71.2
	**non-partnered**	**with someone**	74.4 (13.2)	126	72.1	76.7	63.1 (31.1)	126	57.7	68.5
	** **	**alone**	74.0 (13.6)	1,167	73.2	74.8	67.3 (32.0)	1,168	65.4	69.1

Note. Values are adjusted for gender and age. CM = Chronic Morbidity;

^a ^n varies due to missing values in the outcome measures;

^b^ 95% Confidence Interval.

**Table 4:** Results of the ANCOVAs for mental health and role functioning.

Variable/Effect	Mental Health			Role Functioning		
	df	F	p-value	df	F	p-value
**Corrected Model**	13	141.3	< .001	13	142.4	< .001
**Intercept**	1	8,268.1	< .001	1	1,423.0	< .001
**Age**	1	320.2	< .001	1	106.6	< .001
**Gender**	1	484.6	< .001	1	63.3	< .001
**Health Status**	2	46.5	< .001	2	201.1	< .001
**Relationship Status**	1	81.6	< .001	1	18.2	< .001
**Living Arrangement**	1	15.7	< .001	1	0.5	.50
**Health Status x Relationship Status**	2	2.0	.14	2	0.5	.61
**Health Status x Living Arrangement**	2	0.9	.40	2	0.4	.67
**Relationship Status x Living**	1	14.8	< .001	1	4.4	.04
**Arrangement**						
**Health Status x Relationship Status x**	2	0.1	.87	2	3.8	.02
**Living Arrangement**						
**Error**	25,195			25,164		
**Total**	25,209			22,178		

### Hypothesis 1: Association of chronic morbidity with psychosocial health

There was a significant main-effect for health status on mental health, F(2, 25,195) = 46.5, p < .001, and role functioning, F(2, 25,164) = 201.1, p < .001. As expected, individuals with a single chronic morbidity, and especially those with multiple chronic morbidity, had worse mental health, d_healthy-singleCM_ = 0.09; d_healthy-multipleCM_ = 0.16, and worse role functioning, d_healthy-singleCM_ = 0.14; d_healthy-multipleCM_ = 0.32, than healthy individuals.

### Hypothesis 2: Moderating effect of relationship status

Even though there was a significant main-effect of relationship status indicating that partnered individuals had better mental health, F(1, 25,195) = 81.6, p < .001, d = 0.12, and better role functioning, F(1, 25,164) = 18.2, p < .001, d = 0.06, than non-partnered individuals, relationship status did not significantly modify the effect of health status on mental health, F(2, 25,195) = 2.0, p = .14, and role functioning, F(2, 25,164) = 0.5, p = .61. Thus, the second hypothesis was not confirmed.

### Hypothesis 3: Moderating effect of relationship status and living arrangement

**Fig 1:**
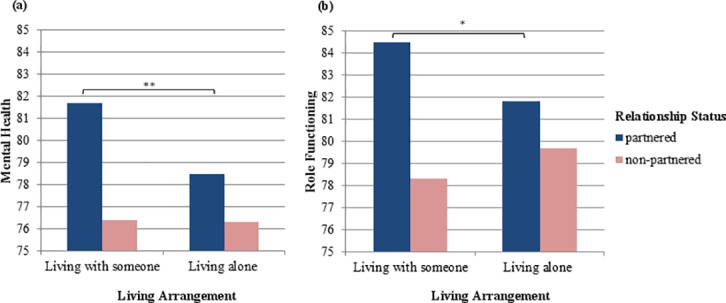
Interaction-effect of relationship status and living arrangement. Note. The y-axes display estimated mean values of (a) mental health and (b) role functioning adjusted for gender and age; * p = .04; ** p < .001.

**Fig 2:**
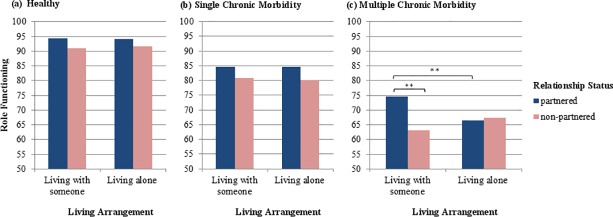
**Interaction-effect of relationship status and living arrangement for (a) healthy individuals, individuals with (b) single and (c) multiple chronic morbidity.** Note. The y-axes display estimated mean values of role functioning adjusted for gender and age; ** p < .001.

The interaction of relationship status and living arrangement was significant for mental health, F(1, 25,195) = 14.8, p < .001, and role functioning, F(1, 25,164) = 4.4, p = .04. Simple effects analyses showed that living arrangement had an effect only in partnered individuals, with those sharing a home with a partner reporting better mental health, F(1, 25,195) = 34.9, p < .001, d = 0.24, and better role functioning, F(1, 25,164) = 4.4, p = .04, d = 0.09, than partnered individuals living alone, Fig 1. This interaction of relationship status and living arrangement was further qualified by health status with respect to role functioning, F(2, 25,164) = 3.8, p = .02, but not mental health, F(2, 25,195) = 0.14, p = .87. Simple effects analyses showed that the living arrangement had an effect on role functioning only for partnered individuals with multiple chronic morbidity, F(1, 25,164) = 10.9, p < .001. In accordance with our hypothesis, partnered individuals with multiple chronic morbidity had better role functioning when sharing a home with their partners as opposed to living alone, d = 0.26, Fig 2. As expected, non-partnered individuals with multiple chronic morbidity did not benefit from sharing a home with others, F(1, 25,164) = 2.0, p = .16; although not statistically significant, it appeared to be related to worse role functioning than living alone, d = 0.13. When living alone, partnered and non-partnered individuals with multiple chronic morbidity did not differ in their role functioning, F(1, 25,164) = 0.08, p = .78, indicating that the benefit of having a partner depends on sharing a home. When sharing a home, partnered individuals with multiple chronic morbidity had significantly better role functioning than non-partnered individuals within the same health status group, F(1, 25,164) = 16.4, p < .001, d = 0.36. These findings suggest that having a partner reduces impairment of role functioning in individuals with multiple chronic morbidity, but only when these individuals are living with that partner. Individuals with multiple chronic morbidity who are partnered but in a LAT-relationship or who are non-partnered (both living alone or living with someone) have impaired role functioning.

## Discussion

This study has shown that middle-aged and older individuals with single and especially multiple chronic morbidity have impaired role functioning compared to healthy individuals. Regardless of their health status, individuals reported better mental health if they had a partner with whom they shared a home. In individuals with multiple chronic morbidity, living with a partner was beneficial in terms of role functioning. In other words, living together with a partner seemed to mitigate the adverse effects of multiple chronic morbidity. Having a partner who lives somewhere else or being non-partnered while living with someone else, did not mitigate these adverse effects.

Consistent with previous studies, we found that disease burden increases with increasing number of morbidities within the same individual [4,10,28] and that this effect is mainly reflected in impaired role functioning rather than mental health [5,6,28–30]. The relatively small effects on mental health might be a consequence of focusing on physical chronic diseases (e.g., arthritic disease, diabetes, cardiovascular disease). In contrast to psychiatric conditions (i.e., depression or anxiety disorder), physical conditions have been found to be mainly negatively associated with physical functioning and not mental health [23,29]. In addition, some authors argue that patients undergo a psychological adaptation process that sustains mental health despite chronic morbidity, e.g., [31,32]. This idea is supported by studies showing that the association of chronic morbidity with mental health declines over time, while the association with impaired physical functioning appears permanent [6,33]. This indicates that patients with physical chronic morbidity adapt mentally to their health condition but remain inflicted with impaired physical and role functioning, i.e., problems performing their daily life activities such as work or hobbies.

Contrary to our second hypothesis, the adverse effects of single and multiple chronic morbidity on mental health and role functioning did not differ between partnered and non-partnered individuals. However, irrespective of having chronic morbidity, sharing a home was beneficial for partnered individuals but it was not beneficial for non-partnered individuals. This effect was most evident for mental health and less so for role functioning. This is in line with the finding that the social support provided by a partner benefits mental health outcomes such as feelings of happiness, satisfaction with life, and mood [14].

When taking health status into account, partly confirming our third hypothesis, only effects for role functioning were found and only in the most severe disease group: individuals with multiple chronic morbidity had better role functioning when having a partner they also shared a home with. Individuals living alone, both partnered (LAT-relationship) and non-partnered individuals, did not differ in their role functioning, indicating that the benefit of having a partner depends on sharing a home. Sharing daily life and frequent proximity may be key factors to the benefits derived from being partnered. However, sharing a home did not help to improve functioning for non-partnered individuals with multimorbidity. Furthermore, non-partnered individuals with multiple chronic morbidity who shared a home had the poorest mean level of role functioning out of all of the groups. This group was largely female (77%), on average 56.4 years of age, and the majority reported living with their children (78.6%). Similarly, older women living with someone other than a partner [19] and specifically those living with children [18] have been found to have impaired mental health and physical functioning. Following the reasoning of Hughes and Waite [18], the poor functioning of this subgroup in our sample may be due to an imbalance of demands and resources, that is, having caregiving responsibilities towards children combined with a high disease burden (multimorbidity) and a lack of partner support.

The major strength of this study is its large, broadly representative sample [34] and its consideration of a great number of chronic morbidities in comparison to a healthy control group. The selection of chronic conditions was based on the impact on daily life and, wherever possible, we included only severe diseases that required treatment. By including two outcome measures, we were able to demonstrate that an individual’s health status was particularly related to role functioning while relationship status and living arrangement were particularly related to mental health. Last, we demonstrated the importance of including relationship status as well as living arrangement when analyzing vulnerability to poor role functioning. Both factors are essential indicators in determining whether individuals need additional support when they are facing multiple chronic conditions.

In addition to these strengths, the outcomes of our study should be interpreted in light of some limitations. First, because we analyzed cross-sectional data, inferences on causality cannot be made. However, the RAND-36 assessed mental health and role functioning in the previous four weeks, while the health conditions probably developed months or years earlier. In addition, previous studies found that chronic morbidity is longitudinally associated with impaired physical functioning [33,35,36] while other studies indicate that support is an underlying mechanism explaining the mental health benefits of having a partner [14]. Thus, it seems likely that chronic disease can impair psychosocial health and support of a partner can mitigate that effect.

Second, the categorization of subgroups was rather crude. Health status was assessed by self-report and multimorbidity was operationalized as disease count. However, participants with inconsistent answers and patients with diseases of unknown and varying severity or treatment were excluded from our dataset. Sensitivity analysis revealed that our findings are robust across disease selection. Analysis without high cholesterol or hypertension considered as chronic morbidities (n = 21,202) largely confirmed our results: only the two-way interaction of relationship status and living arrangement failed to reach significance for the outcome of role functioning, p = .075, yet pointed in the same direction. Furthermore, relationship status was defined as a simple dichotomy. While this might be less relevant for partnered individuals [14], especially as we took living arrangement into account, it seems likely that never-married individuals function differently than those who have been recently divorced or widowed [11,12].

As our study indicates that individuals with multiple chronic diseases benefit from having a partner they share a home with, future studies should investigate the prerequisites of this positive effect. As the couple ages, it is likely that both partners will suffer from single or multiple chronic morbidity. One could imagine that living with a partner who has serious health problems could lead to erosion of spousal support. It should be studied whether the partner’s health status modifies the benefit of having a partner, that is, whether the benefit depends not only on living together but also on the health of the partner. Recent research has demonstrated that a partner’s health status is positively associated with perceived support and relationship quality [22], suggesting that having a healthy partner potentially yields the most benefits when one is ill. A dyadic study could provide more insight into how couples cope in the circumstance that both partners of a couple suffer from chronic morbidity and whether living arrangement affects their adjustment.

Our study has important implications. As the aging of the population is ongoing and relationships and living situations are increasingly diverse among the older age groups, society can expect to be confronted with a growing proportion of middle-aged and older individuals with impaired functioning. The health care system should target individuals with multiple chronic morbidity and particularly those who are non-partnered or have a partner but do not live together. Some patients may benefit from interventions that encourage physical activities [37,38] while others might need support in adapting to their physical limitations by learning how to plan their daily lives accordingly and to formulate realistic daily goals. This is particularly important for middle-aged individuals with the prospect of a long illness duration while still having occupational demands or caregiving responsibilities (e.g., for children). Even though our study did not find major impacts of chronic morbidity on mental health, the possibility of the need for psychological support in some patients should not be ignored.

In conclusion, multimorbidity imposes a large disease burden on middle-aged and older individuals, mainly reflected in impaired role functioning. In individuals with chronic morbidity, not only the number of chronic diseases but also sociodemographic variables (whether one has a partner and shares a home) determine vulnerability to impaired role functioning. In the group of individuals with multiple chronic morbidity, having a partner benefits role functioning only when partners share a home. Individuals with a high disease burden who are non-partnered (particularly when sharing a home) or who do have a partner but live alone (LAT-relationship) are at risk for impaired role functioning and should be targeted to sustain or improve physical functioning.
